# Th17 Cells in Viral Infections—Friend or Foe?

**DOI:** 10.3390/cells10051159

**Published:** 2021-05-11

**Authors:** Iury Amancio Paiva, Jéssica Badolato-Corrêa, Débora Familiar-Macedo, Luzia Maria de-Oliveira-Pinto

**Affiliations:** Laboratory of Viral Immunology, Fundação Oswaldo Cruz, Rio de Janeiro 21040-900, Brazil; iury.iap@gmail.com (I.A.P.); jessicabadolato04@gmail.com (J.B.-C.); deborafamiliar@gmail.com (D.F.-M.)

**Keywords:** Il-17, Th17 cells, viruses

## Abstract

Th17 cells are recognized as indispensable in inducing protective immunity against bacteria and fungi, as they promote the integrity of mucosal epithelial barriers. It is believed that Th17 cells also play a central role in the induction of autoimmune diseases. Recent advances have evaluated Th17 effector functions during viral infections, including their critical role in the production and induction of pro-inflammatory cytokines and in the recruitment and activation of other immune cells. Thus, Th17 is involved in the induction both of pathogenicity and immunoprotective mechanisms seen in the host’s immune response against viruses. However, certain Th17 cells can also modulate immune responses, since they can secrete immunosuppressive factors, such as IL-10; these cells are called non-pathogenic Th17 cells. Here, we present a brief review of Th17 cells and highlight their involvement in some virus infections. We cover these notions by highlighting the role of Th17 cells in regulating the protective and pathogenic immune response in the context of viral infections. In addition, we will be describing myocarditis and multiple sclerosis as examples of immune diseases triggered by viral infections, in which we will discuss further the roles of Th17 cells in the induction of tissue damage.

## 1. Introduction

An efficient immune response against pathogens is essential for their elimination by the host. At the same time, it is essential for homeostasis that the immune system can tolerate its own components, as well as other external antigens, such as those of commensal bacteria and those present in food. Elucidation of the mechanisms that allow the adaptive immune system to perform these tasks efficiently remains a major challenge for science. Effective control of viral infection requires the elimination of infected cells to limit the production and spread of the virus, as well as to establish a specific immune memory directed against viral antigens. Studies have shown that, although Th17 cells appear to be crucial in suppressing certain viral infections, they are also implicated in inducing harmful conditions in this context, since Th17 cells mediate tissue damage and orchestrate chronic tissue inflammation in different target organs.

## 2. Th1/Th2 Paradigm and Discovery of Th17 Cells

In the 1980s, with the advent of T lymphocyte cloning technologies and antibody neutralization assays, Mosmann et al. identified two subpopulations of T lymphocytes [1]: T helper 1 (Th1) lymphocytes, which favored IL-2 and IFN-γ production, and T helper 2 (Th2) lymphocytes, which favored IL-4 production. At the same time, Coffman et al. sought to understand how IgE was produced [2]. Together, Mosmann and Coffman evaluated the Th1 and Th2 subpopulations for IgE production and saw that supernatants from Th2 cell cultures were able to induce robust IgE production by B cells, but Th1 cells did not have this capacity [3]. These data were confirmed later when it was seen in neutralizing assays that IFN-γ inhibited IgE production by B cells, while IL-4 induced it [4]. A year later came the demonstration that Th1 cells participate in delayed-type hypersensitivity, which was not observed in Th2 cells [5].

These and other findings enabled the description of the Th1/Th2 paradigm [6] and characterized Th1 cells as high producers of IL-2 and IFN-γ that were involved in the cell-mediated immune response against intracellular pathogens and as important inducers of IgG from B cells. Th2 cells have been characterized as high IL-4 producers, are the main mediators of IgE production by B lymphocytes and are involved in allergic reactions, formation of eosinophilic infiltrate and elimination of extracellular pathogens, especially helminths. Th1/Th2 regulation is done in an autocrine manner that depends on the cytokines secreted. At the same time, it can also promote or inhibit differentiation of one subpopulation or another [6].

Discovery of the Th1/Th2 paradigm brought knowledge about atopic diseases, tolerance to autoantigens and susceptibility or resistance to pathogens [7]. However, the mechanisms involved in autoimmune diseases remained to be explained. Data supporting the role of Th1 cells in inducing experimental autoimmune encephalomyelitis (EAE), a murine model of multiple sclerosis that affects the central nervous system in humans, were not entirely convincing [8]. Administration of IFN-γ in mice and rats susceptible to EAE was shown to alleviate the symptoms associated with the disease [9] and treatment with IFN-γ blocking antibodies induced the appearance of severe symptoms of EAE [10]. Thus, the role of Th1 cells or their mediators alone was not able to explain the mechanisms of the induction of autoimmune diseases.

Subsequently, in 2000, a new cytokine chain named p19 was discovered, which helped to elucidate issues regarding the Th1/Th2 paradigm and autoimmune diseases [11]. Starting the historical cascade of discoveries, p19 forms heterodimers with the IL-12 p40 chain (IL-12p40), giving rise to the cytokine IL-23. Thus, IL-23 is formed by the heterodimer p19 and IL12p40; IL-12 is formed by the interaction of the p35 and IL-12p40 chain. IL-23 binds to its IL-23R receptor and IL-12Rβ1 (known as the IL-23R complex), while IL-12 interacts with the IL-12Rβ1 and IL-12Rβ2 chains [12]. Production of IL-12 is mainly induced through activation of dendritic cells (DC) by microbial products and IL-23 through activation of DC by prostaglandin E2 and adenosine triphosphate and activation via anti-CD40 [13,14]. Aggarwal et al. demonstrated that IL-23 induced the production of IL-17 by CD4 T lymphocytes, and that this cytokine was expressed neither by Th1 nor by Th2 [15]. Cua et al. demonstrated that IL-23 was able to promote expansion of IL-17-producing T cell clones and that the adoptive transfer of these cells to wild-type mice induced EAE [16]. Based on these and other studies, the scientific community proposed that IL-17-producing T cells would belong to a subpopulation of Th lymphocytes distinct from Th1 and Th2, and this was called Th17 [17,18].

Th17 cells are classified as a subpopulation of CD4 T lymphocytes that has unique effector functions and specific transcription factors that control differentiation and function [19]. They have been identified as producing mainly the cytokine IL-17A, but also IL-17F, IL-21, IL-22, IL-26, CXCL8 (IL-8) and CCL20 [20]. The transcription factor retinoic acid-related (RAR) orphan gamma receptor t (RORγt) has been identified as the main inducer of differentiation of Th17 cells in mice [21]. In humans, the transcription factor responsible for the differentiation of Th17 is the RAR-related orphan gamma receptor (RORC2) [22].

**Figure 1:**
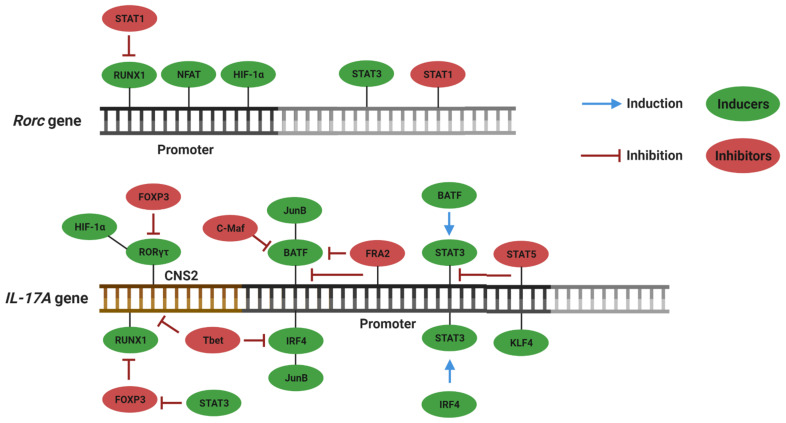
Transcription factors that positively and negatively regulate the differentiation of naive CD4 T cells in Th17. Nuclear factor of activated T cells (NFAT) and NF-κB can activate the *rorc2* promoter in humans. Furthermore, Runt-related transcription factor 1 (RUNX1), which is linked to the CNS2 region of the *il17a* gene, potentiates expression of this gene. Alternatively, RUNX1 can directly bind to the gene that encodes RORγt and induces Th17. Another important element is hypoxia-inducible factor-1α (HIF-1α), which binds and acts as a co-activator for RORγt. It is important to note that the loci *il21*, *il22* and *il23r* contain binding regions for basic leucine zipper ATF-like transcription factor (BATF) and interferon regulatory factor 4 (IRF4), thus suggesting that these elements are involved in transcription of other genes relating to the Th17 subpopulation. BATF also forms heterodimers with the transcription factor jun-B (JunB) and binds to the promoter of *il17a*, as well as kruppel-like factor 4 (KLF4). Therefore, BATF is involved in inducing IL-17A expression [24]. Furthermore, aryl hydrocarbon receptor (AhR) has also been shown to be important in inducing Th17 [26]. BATF and IRF4 appear to act in the process of inducing differentiation in Th17 through inducing signal transducer and activator of transcription 3 (STAT3) and leading to changes in chromatin, in a way that allows exposure of binding sites at the *il17a* locus [24]. STAT3 directly regulates the IL-17A, IL-17F and IL-23R genes and binds and regulates the expression of BATF and IRF4 [27]. STAT3 also increases the expression of RORγt and decreases FoxP3 expression (Forkhead box P3), a master regulator of development and functioning of regulatory T cells, in addition to interacting with the promoter of *il17a* and *il17f* [21]. The Th17 differentiation process is believed to take place in three main transcriptional steps. First, the *stat3*, *irf4*, *batf*, *il21* and *il23r* genes are induced. This causes transcription of Rorc to begin, which ultimately induces expression of cytokines from the Th17 profile while inhibiting expression of cytokines from other profiles [24]. On the other hand, there are transcription factors that negatively regulate differentiation of naive CD4 T lymphocytes in Th17, especially c-Maf, which attenuates expression of genes involved in pro-inflammatory functions, such as *batf*, *rorα*, *runx1*, *ccr6*, *il1r1* and *Tnf*, among others, and induces the expression of genes relating to control of the immune response, such as *il10* and *ctla4* [24]. Another controller of the immune response with a Th17 profile is Fos-related antigen 2 (Fosl2, also FRA2), which competes for the binding site and inhibits BTF4 expression [28]. Other known inhibitors of Th17 differentiation are STAT1, which binds to the Rorc locus, and STAT5, which inhibits expression of IL-17A through binding to the *il17a* locus and removing STAT3 molecules bound therein [24]. T-box transcription factor (T-bet), which is related to differentiation in Th1, and FoxP3, which is linked to induction of Tregs, also act by negatively regulating Th17. T-bet inhibits expression of IRF4 and prevents the binding of RUNX1 to RORγt [29,30]. FoxP3, on the other hand, binds directly to RORγt and RUNX1 and inhibits the differentiation of Th17 cells [31,32]. The following other factors have also already been described as Th17 inhibitors: Twist Family BHLH transcription factor 1 (TWIST1), peroxisome proliferator-activated gamma receptor (PPARγ), E-twenty six 1 (ETS1), E74-like factor 4 (ELF4), inhibitor of DNA-binding (ID3) and early growth response gene (EGR2) [33].

RORs belong to a superfamily of ligand-regulated transcription factors. Interaction with the ligands allows recruitment of accessory proteins, followed by transcription of target genes. The ROR family is composed of three members: RORα, RORβ and RORγ. The *ror* genes encode different protein isoforms, among which RORα4 and RORγt are the isoforms expressed in cells of the immune system [23]. RORγt binds to the conserved noncoding sequence 2 (CNS2) region of the *il17a* gene and induces its transcription [24]. Because the RORα4 and RORγτ binding sites are highly similar, it is believed that activation of the transcription of *il17a* by both isoforms occurs through similar mechanisms [25]. Although RORγt and RORC2 are the main transcriptional inducers of Th17, other inductors have been identified, as described in the scheme shown in Figure 1.

The research groups of Romagnani, Napolitani and Farber demonstrated that CCR6 is expressed by IL-17-producing T cells, although not all CD4 + CCR6 + T cells produce IL-17A [34,35,36]. CCR6 is a chemokine receptor associated with protein G, and its only ligand is CCL20, which is produced in high concentration by Th17. Expression of CCR6/CCL20 ensures chemotaxis of Th17 to a wide variety of tissues, such as the intestine, central nervous system and skin [37,38,39]. Furthermore, CD161, which had previously been identified as a marker for natural killer cells (NK) and NKT, has also been shown to be a marker for Th17 precursor cells [40].

Through evaluating the expression of CCR4 and CXCR3 in CCR6 + cells, two functionally distinct subpopulations of Th17 lymphocytes can be identified. CCR6 + CCR4 + CXCR3- cells identify Th17 cells themselves, express RORC and have IL-17A as their main effector cytokine [35]. CCR6 + CCR4 - CXCR3 + cells identified in the Th1Th17 subpopulation produce both IL-17A and IFN-γ [35,41,42,43]. Both subpopulations express IL23R, IL-1R, CD26 and CD161 on their surface [44]. Th1Th17 cells have characteristics of both Th1 and Th17 cells while expressing RORC and the characteristic transcription factor of Th1, i.e., T-bet [45]. Unlike Th17, Th1Th17 cells express the IL-12 receptor (IL-12R), which is a potent Th1 inducer [46]. Additionally, Th1Th17 cells have been identified as the main subpopulation of CD4 T lymphocytes in the inflammatory infiltrate of autoimmune diseases [44,47].

**Figure 2:**
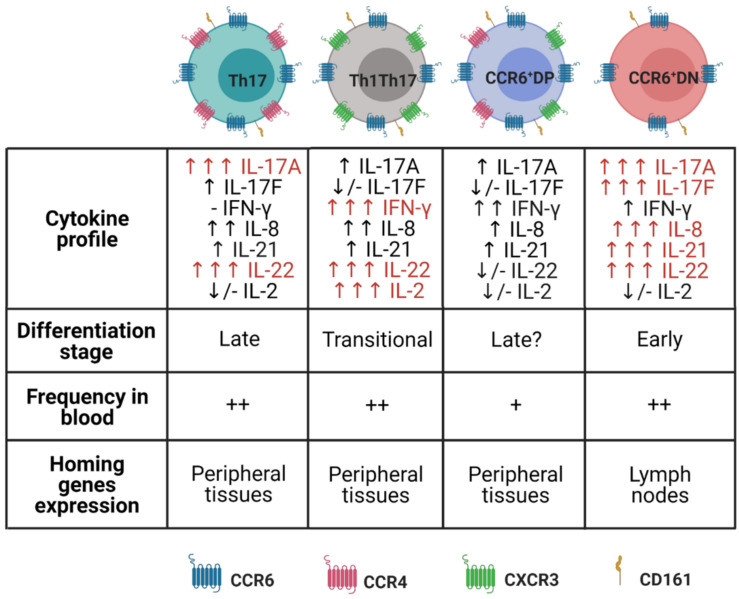
Different subpopulations of naive CD4 T lymphocytes in Th17 cells. This schematic drawing demonstrates the four different types of Th17 cells with the expression markers, secreted cytokine profiles, differentiation state, homing and frequency in blood that are characteristic of each subpopulation [48]. ↑↑↑ = high production; ↑↑ = medium production; ↑ = low production; ↓/- = very low/no production. ++ = higher frequency; + = lower frequency.

Ancuta et al. characterized two other subpopulations of Th17 lymphocytes. They were called CCR6 + Double-Negatives (CCR6 + DN; CXCR3 - CCR4 -) and CCR6 + Double-Positive (CCR6 + DP; CXCR3 + CCR4 +). The level of IL-17A production after stimulus via TCR by CCR6 + DN is similar to that of Th17. CCR6 + DP is similar to Th1Th17 in this regard. These two subpopulations are capable of secreting IFN-γ. In the same study, analyses on the broad transcription profile of the genome made it possible to observe further differences between these two subpopulations. CCR6 + DN express chemotaxis markers for lymph nodes, such as CCR7 and CXCR5, along with high levels of STAT3 and IL-17F mRNA, which are both related to early stages of differentiation in Th17. Furthermore, these express genes that are related to cell survival and proliferation, such as *lef1*, *myc*, *terc* and *nanog*, which are characteristic of stem cells. On the other hand, CCR6 + DP, similar to Th17, expresses high levels of LMNA, a marker of senescence. It was suggested that CCR6 + DN represents an early stage of differentiation, compared with Th17 and CCR6 + DP [48]. Based on this work, we have compiled Figure 2 to aid in understanding the different subpopulations of Th17 cells.

## 3. Plasticity and Effector Abilities of Th17 Modulated by Inflammatory Cytokines

Differentiation of T lymphocytes is a complex orchestrated process implemented by transcription factors that promote expression of genes that induce effector profiles and, at the same time, inhibit expression of genes relating to other distinct profiles. It is believed that genes relating to other Th subpopulations would be expressed at very low levels. This capacity for induction would lead to functional reprogramming of already-differentiated Th cells, which would be a remarkable phenomenon of functional plasticity [49]. Th17 cells are known for their high capacity to acquire phenotypic and functional characteristics of other subpopulations of CD4 T lymphocytes, such as Th1, Th2, Treg and Tfh (T follicular) [47].

In addition to plasticity, a new subset of non-pathogenic IL-10-producing Th17 cells has recently been discovered. Therefore, non-pathogenic IL-10 + Th17 cells do not induce tissue inflammation and inhibit autoimmune inflammation [50]. The differentiation of non-pathogenic or pathogenic Th17 cells depends on the cytokine milieu in which the naïve T cells are present. Naive T cells treated with TGF-β1 and IL-6 can promote the generation of non-pathogenic Th17 cells [51], a process that can be nullified by exposure to IL-23, resulting in pathogenic Th17 cells [52]. The gene expression profiles of in vitro polarized Th17 cells identified a differential expression of 233 genes between the two Th17 subsets. Pathogenic Th17 cells express more effector molecules, including pro-inflammatory cytokines/chemokines, such as CXCL3, CCL4, CCL5, IL-3 and IL-22 and transcription factors, such as Tbx2 and Stat4, while non-pathogenic Th17 cells exhibit positive regulation molecules related to immune suppression, cytokines, such as IL-10, and transcription factors, such as Ikzf3 [53]. Although the characterization of the requirements for the generation of non-pathogenic Th17 cells has advanced, the mechanism underlying the generation of IL-10 + Th17 cells has not yet been fully elucidated [54].

Returning to the remarkable phenomenon of functional plasticity of Th17 cells, acquisition of characteristics of the Th1 profile by Th17 cells has already been demonstrated in mice and in humans, in the presence of IL-12, through decreased expression of RORγt/RORC, IL-17A, IL-17F, IL-22 and CCR6 and increased T-bet and IFN-γ [55,56,57].

However, the plasticity of Th17 is not limited to the Th1 profile alone. Cosmi et al. exposed CD4 T lymphocytes from CCR6 + CD161 + memory, from patients with asthma, to an IL-4-rich microenvironment and demonstrated that these cells started to produce Th2-profile cytokines such as IL-4 and IL-5 and to express the GATA-3 transcription factor. While doing this, these cells maintained their ability to express IL-17A, IL-21 and IL-22 [58]. This profile was called Th2Th17.

Tfh are important for inducing immunoglobulin class change and producing antibodies with high affinity for B cells. Hirota et al. demonstrated that Th17 cells present in the Peyer plates of mice acquired a Tfh lymphocyte phenotype, with increased expression of Bcl-6 (B-cell lymphoma 6 protein), CXCR5, PD1 (programmed cell death-1) and IL-21. Moreover, they were able to induce IgA production from germinal center B lymphocytes [59]. Because Bcl-6 can suppress the expression of T-bet, GATA3 and RORγt, IL-21 is indispensable in relation to differentiation of CD4-naive T cells into Tfh. Hence, these are probably the factors involved in re-differentiation of Th17 in Tfh [59,60].

**Figure 3:**
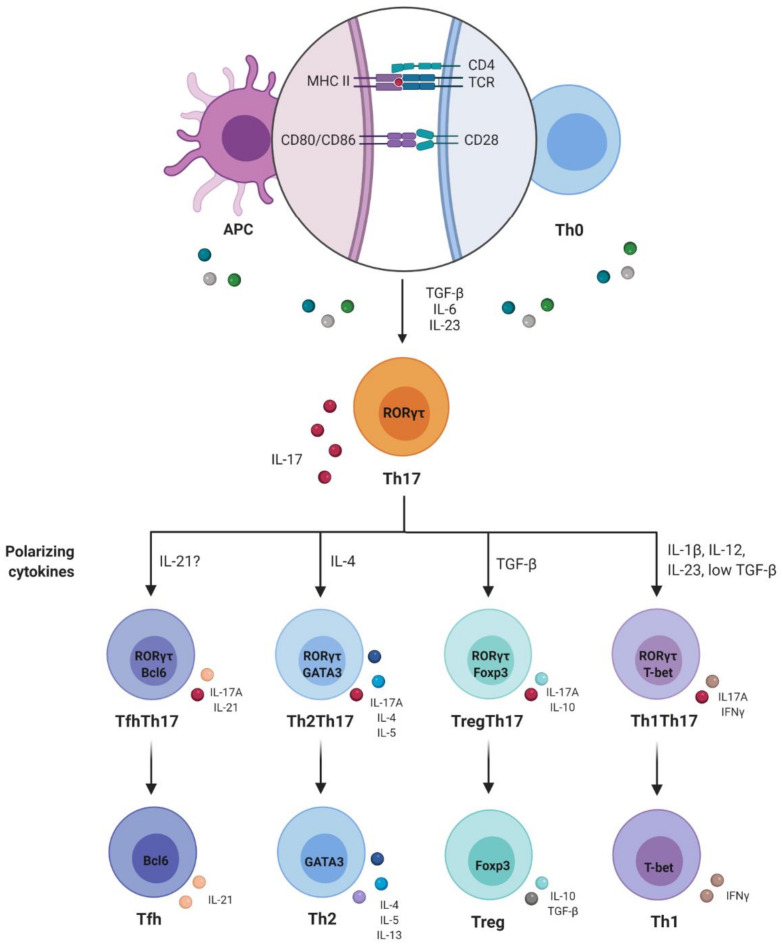
Plasticity of Th17 cells. The plasticity of Th17 cells suggests that this subpopulation has a range of functions and different migration patterns and anatomical locations, and that it may be involved in the protective immune response to a wide variety of infectious agents and autoimmune diseases that affect different organs.

Therefore, differentiation of naive CD4 T lymphocytes into Treg and Th17 cells appears to be interconnected. While TGF-β alone induces FoxP3 expression and differentiation in Treg, differentiation of Th17 occurs in the presence of IL-6 or IL-21 [61,62]. The plasticity between these two profiles has been demonstrated in a series of studies, both in mice and in humans. Production of IL-17 by Treg has already been detected and was found to be associated with decreased expression of FoxP3 and increased expression of RORγt/RORC, induced by IL-1β and IL-23 [63,64]. On the other hand, conversion of Th17 cells to Treg was also seen in a study by Hoechst et al. [65]. Those authors used a co-culture system of monocytes and Th17 lymphocytes and observed that, under these conditions, there was a decrease in the frequency of CD4 + IL17 + T cells, while there was an increase in CD4 + IL17 + FoxP3 + and IL17 - FoxP3 +. This effect was mediated by TGF-β and retinoic acid [65]. The plasticity of Th17 is shown in Figure 3.

The broad spectrum of effector functions of cytokines secreted by Th17 is defined through their action on a wide variety of cells, which may or may not belong to the immune system. As stated earlier, the major cytokine secreted by Th17 is IL17A. In addition, Th17 cells are major producers of IL-17F, IL-21, IL-22, IL-26 and CCL20. To exemplify the wide variety of cells influenced by these mediators, the effect of IL-21 on B cells in inducing their proliferation, changing the isotype class, and amplifying differentiation of the Th17 profile can be highlighted. In addition, IL-17A, IL-17F, IL-22, IL-26 and CCL20 induce the production of inflammatory cytokines and chemokines that are involved in recruitment of granulocytes, especially neutrophils, to inflammation sites. Moreover, they act towards the secretion of antimicrobial peptides by epithelial cells present in barriers, such as skin and mucous membranes [19]. IL-17-producing cells are found in abundance in the oral cavity, gastrointestinal tract, lungs, vagina, and skin. In fact, Th17 cells are known to be indispensable in inducing protective immunity against bacteria and fungi in mucosal tissues because they promote the integrity of the epithelial barrier [44].

IL-17 is a protein of molecular weight 15 kDa. In humans, its gene is located on chromosome 6p12. The IL-17 family of cytokines includes IL-17A, IL-17B, IL-17C, IL-17D, IL-17E and IL-17F. To date, the best-described members are IL-17A and IL-17F, which have about 50% similarity between their amino acid sequences, compared with other members of the family. They can form homodimers through disulfide bonds (IL17A-IL17A) or heterodimer bonds (IL17A-IL17F) [66]. One important difference between these two is that IL-17F is expressed more in the early stages of Th17 differentiation, and this expression decreases as the process takes place [44]. The cytokines IL-17A and IL-17F are produced by a wide variety of cells, including T γδ, T CD8 (Tc17) lymphocytes, NKT, NK cells, mast cells, eosinophils, and neutrophils. Thus, they have an ability to serve as a bridge between innate and adaptive immune responses [66].

The IL-17 receptor family (IL-17R) is composed of five subunits: IL17RA, IL17RB, IL17RC, IL17RD and IL17RE. IL17Rs contain conserved domains in their structure: an extracellular and a cytoplasmic domain. Only the IL17RA subunit contains two cytoplasmic domains linked to SEFIR: the Toll/IL-1R-like loop (TILL) and the distal end domain at the C-terminal. These subunits can form different complexes that will serve as receptors for the cytokines of the IL-17 family. For example, IL-17A and IL-17F exert their function by binding to the receptor complex formed by the IL17RA-IL17RC subunits. IL-17C acts by binding to an IL17RA-IL17RE receptor, and IL-17E acts by interacting with IL17RA-IL17RB. Thus, it appears that the IL17RA subunit is common to all receptor complexes in this family [67].

By stimulating production of CXCL1, CXCL2, CXCL5 and CXCL8/IL-8, IL-17A acts in the recruitment of neutrophils [20]. Production of granulocyte macrophage colony-stimulating factor (GM-CSF) is highly induced by IL-17A. In addition to GM-CSF being a potent inducer of granulocyte production and maturation, the IL-17A-GM-CSF axis is important for the process in which these cells exit from the bone marrow and migrate to inflammatory sites [61]. IL-6 and TNF- are pro-inflammatory cytokines induced by IL-17. As already mentioned, among other functions, IL-6 acts to amplify differentiation in Th17 [68,69].

Hsu et al. demonstrated that IL-17 contributes to the formation of the germinal center in the spleen of mice that produce autoantibodies capable of causing autoimmune disorders. In these mice, they found that there was a higher frequency of Th17 cells in the spleen, compared with wild-type animals [70].

In addition to the role of Th17 in different cell populations, Th17 cells are an important source of cytokines. The cytokine IL-22 is a member of the IL-10 family, which also comprises the cytokines IL-19, IL-20, IL-24, IL-26, IL-28 and IL-29. The main IL-22-secreting cell population is the Th22 subpopulation of CD4 T lymphocytes [71]. Production of IL-22 by Th17 is dependent on the action of IL-23. One of the mechanisms for its inhibition is through the action of TGF-β, which inhibits the expression of IL-23R [31].

Another cytokine of relevance is IL-26. The main source of production of the cytokine IL-26 is activated Th17 lymphocytes. Studies have identified high expression of IL-26 in chronic inflammatory diseases, thus suggesting that IL-26 is a potent pro-inflammatory mediator. Interestingly, IL-26 is expressed by IL-17-producing T cells present in inflammatory infiltrate in the joints, skin, liver, lungs and intestines of patients affected by inflammatory bowel disease [72]. It has been seen that IL-26 secreted by Th17 acts as an antimicrobial factor and also induces the production of type 1 IFN by plasmacytoid dendritic cells via TLR-9 [73]. Naive CD4 T lymphocytes express low levels of IL-26, compared with memory CD4 T lymphocytes, which suggests that this cytokine may be a marker of highly differentiated Th17 cells [72]. The mechanisms for the induction of this cytokine have not yet been fully described. It has been shown that IL-1β and IL-23 induce the production of IL-26 and that this, in turn, induces the production of IL-17 and IL-23 by CD4 T lymphocytes [74,75]. Thus, this positive feedback loop may be crucial in maintaining the inflammatory profile of lymphocytes in the cellular infiltrates found under the pathological conditions previously mentioned.

## 4. Th17 Cells in Viral Infections

Studies have shown that IL-17, while appearing to be crucial for controlling viruses, also induces deleterious conditions in certain viral infections. We will now discuss the involvement of Th17 in some viral infections and these two possible situations. Tissue damage can be caused by direct viral replication or by an immunopathological response. In this sense, it has already been described that inflammation induced by Th17 cells can increase immunopathology and tissue damage observed in diseases such as multiple sclerosis and viral myocarditis, both of which in viruses have already been described as triggering agents.

### 4.1. Severe Acute Respiratory Syndrome Coronavirus 2 (SARS-CoV-2)

COVID-19 is a disease that emerged as a pandemic in March 2020 [76]. There is still no specific treatment for the disease and only management of patients’ symptoms is performed. COVID-19 is caused by the severe acute respiratory syndrome coronavirus 2 (SARS-CoV-2). Some infected individuals develop an acute respiratory disorder syndrome (ARDS) characterized by pulmonary edema and lung failure, along with damage in other organs such as the liver, heart and kidneys [77,78]. These symptoms are related to a “cytokine storm” characterized by high circulating levels of pro-inflammatory mediators. These include IL-1β, IL-6, IL-17 and GM-CSF, which are cytokines involved in the effector function and/or in differentiation of Th17 [78]. In a systematic review and meta-analysis on eight related studies, Coomes and Haghbayan concluded that patients with severe COVID-19 have circulating levels of IL-6 almost three times higher than observed in mild patients and that IL-6 blockade through use of a receptor antagonist has led to better prognosis for treated individuals [79]. Especially in the case of COVID-19, it is worth mentioning that IL-6 is an inducer of differentiation of Th17, while IL-17 induces the secretion of IL-6 [79].

Gil-Etayo et al. proposed very interesting but controversial data in relation to other studies. These authors argue in a very interesting way that, in fact, it is not the total percentage of each Th subset that should be seen as a prognostic factor, but rather the degree of Th activation. In this study, the Th2 cells were more associated with the poor prognosis, since the authors found higher percentages of senescent Th2 cells in patients who died than in those who survived. On the other hand, no significant differences were observed either in the percentage of Th1 or Th17, or in the degree of activation of these cells in the groups of patients with COVID-19 [80].

More recent studies have advanced in terms of a better definition of the immunopathogenic role of Th17 cells in COVID-19. Thus, Meckiff et al. presented single-cell transcriptomic analysis of >100,000 viral antigen-reactive CD4 + T cells from COVID-19 hospitalized patients compared to non-hospitalized patients. Briefly, two clusters were relatively underrepresented for SARS-CoV-2-reactive CD4 + T cells, which were both enriched for Th17 signature genes and highly enriched for cells expressing IL17A and IL17F transcripts. Moreover, polyfunctional Th1 and Th17 cell subsets were underrepresented in the repertoire of SARS-CoV-2-reactive CD4 + T cells compared to influenza-reactive CD4 + T cells [81]. Until this moment, the functional relevance of an impaired Th17 response in COVID-19 is not clear.

However, Zhao et al., in search of a greater understanding of the role of Th17 cells, investigated the profile of immune cells in bronchoalveolar lavage fluid and blood collected from severe COVID-19 patients and patients with bacterial pneumonia not associated with viral infection. The authors identified clonally expanded tissue-resident memory-like Th17 cells (Trm17 cells) in the lungs, even after viral elimination. These Trm17 cells were characterized by a potentially pathogenic cytokine expression profile of IL17A and CSF2 (GM-CSF). Interactome analysis suggests that Trm17 cells may interact with pulmonary macrophages and cytotoxic CD8 + T cells, which have been linked to disease severity and lung damage. Moreover, elevated IL-17A and GM-CSF protein in the serum of patients with COVID-19 have been associated with a more severe clinical course. Collectively, our study suggests that lung Trm17 cells are a potential orchestrator of severe COVID-19 hyperinflammation [82].

In addition, Toor et al. published a very interesting review about potential therapeutic approaches to treat patients with COVID-19. This includes Th17 blockers as an important therapy for improving T cell antiviral responses against SARS-CoV-2 [83,84].

Another much more recent study found a high frequency of central memory CD4 + CCR6 + Th17 subpopulations and high circulating IL-17 levels in the critically ill patients with COVID-19. In the set of data obtained by the authors, critical COVID-19 was characterized by a Th17-mediated response and dysfunctional response associated with IFN-γ, indicating an impaired ability to mount antiviral responses during ARDS [85].

Currently, several reviews are being produced containing many other approaches on the role of Th17 cells in severe cases of COVID-19, and these should be consulted by interested readers [86,87].

In fact, the “cytokine storm” is associated with gravity and is a major cause of death by COVID-19 [88]. Increased levels of circulating pro-inflammatory mediators seen in severe cases, including those involved in the Th17 effector function, could indicate the involvement of Th17 cells in a poor clinical outcome. In addition, recent approaches have shown that the specific repertoire of SARS-CoV-2-reactive CD4 + T cells and Trm17 cells in the lungs has been associated with severe COVID-19 hyperinflammation. This has allowed several authors to encourage the use of Th17 blockers as therapy. However, studies have been carried out, and there are also contradictory results regarding the participation of Th17 in immunopathology. In addition, there is insufficient data in the literature to define which types of Th17 cells would be involved in the pathology, whether pathogenic Th17 cells or conventional Th17 cells.

### 4.2. Influenza Virus

The protective role of B cells during viral infections is mainly mediated by an effective humoral response, with production of specific neutralizing antibodies against the infectious agent [89]. Few studies have addressed the role of IL-17 in modulating B cell activity during viral infections. One of the studies most cited in the literature is that of Wang et al., who assessed the role of IL-17 in infection with the H5N1 influenza virus. In their study, infected *il17*^-/-^ mice showed greater susceptibility to infection by the virus and lower survival rate than wild-type mice. Histological analysis showed a lower number of B cells (B220+) in the lungs of *il17*^-/-^ animals than in wild-type mice, which suggests that IL-17 plays a crucial role in the recruitment of B cells into the lungs after H5N1 infection and that this phenomenon is dependent on chemokine ligands and receptors such as CXCL13 and CXCR5 in B cells [90].

In infection with the H1N1 influenza virus, the same research group observed that infected *il17a*^-/-^ mice had a lower survival rate, greater tissue damage and greater viral load in lung tissue than wild-type mice. Intranasal administration of H1N1 led to high production of IgM antibodies in wild-type mice, while in knockout mice, the presence of IgM was profoundly reduced. This was probably related to decreased capacity of B cells of the profile B1 to produce this immunoglobulin. The result of this was inefficient viral clearance. Through investigating the mechanisms that would lead to this, these authors found that IL-17 was essential for inducing the expression of B lymphocyte-induced maturation protein 1 (Blimp-1) and NF-κB by lung B1 cells, which are essential factors for differentiation and production of IgM by these cells [91]. McKinstry et al. demonstrated that *il10*^-/-^ mice challenged with lethal doses of the H1N1 influenza virus had a higher survival rate and less weight loss than wild-type mice [92]. Analysis on the lungs of these animals showed that absence of IL-10 led to increased levels of IL-6, IL-17 and IL-22 but did not influence the levels of IFN-γ, IL-12 and TNF-α. Transfer of Th17 or Th1 cells isolated from previously immunized mice to naive mice demonstrated that among animals that received Th17, the survival rate was similar to that observed among non-infected animals. The mice that received Th17 showed less alteration of respiratory parameters and less viral load than those that received Th1 cells [92]. Other very interesting data have been published by McKinstry et al. The authors showed in *il-10* deficient mice exhibit increased survivability compared to wild-type mice when challenged with lethal doses of H1N1 or H3N2. This protective response was correlated with a strong Th17 response, in addition to a strong Th1 response and increased expression of various cytokines associated with Th17 in the lungs of these animals during the peak of infection. Thus, the expression of IL-10 inhibits the development of Th17 responses during influenza infection, and this is correlated with impaired protection during the primary, but not secondary, high-dose challenge [92].

As far as we discussed, IL-17 and Th17 cells appear to play an immunoprotective role in influenza, mainly because of their ability to recruit B cells to the lungs, which is the most affected organ. The last study we discussed showed that IL-10 expression inhibits the development of Th17 responses during influenza infection and that this is correlated with impaired protection. To date, studies agree more on the immunoprotective role of Th17 in influenza infection, but it is not known whether, for example, non-pathogenic Th17 cells are differentiated in these cases.

### 4.3. Herpes Simplex Virus (HSV)

CD4 T cells, especially Th1 cells, play a fundamental role in protecting against HSV [93]. Here, it is worth mentioning the study by Anipindi et al. They found that *il-17a*^-/-^ mice were more susceptible to death when infected by HSV-2. This was thought to be because the DCs of the vagina of these animals were not able to promote differentiation of Th1 cells, compared with wild-type mice, which would suggest that IL-17 plays a crucial role in the ability of DCs in these animals to induce a Th1 response [94]. Additionally, in this context, another study showed that IL-17A-induced protection is important with regard to secondary HSV-2 infection, since *il17a*^-/-^ mice that were re-exposed to the virus were more susceptible to virus spread, morbidity and mortality than were knockout mice in primary infection [95].

Very few studies have been done on the role of Th17 in HSV infection in humans. We highlight the recent study by Mei et al. on recurrent herpes labialis (RHL), which is a common skin disease, often caused by the HSV-1 in patients. The ratio of Th17/Treg cells in the peripheral blood of RHL patients was significantly increased compared to healthy volunteers. As well as an increase in the levels of GM-CSF, IL-4, TGF-β, IL-12, IL-10, IL-17F and TNF-α, higher expression of IL-4, IL-10 and TGF-β were detected in RHL patients compared to healthy volunteers, indicating an imbalance of Th17/Treg cells in RHL that is likely to be an important factor in the occurrence, development and recovery of RHL [96].

Unfortunately, few studies address the involvement of Th17 cells in HSV infection. Based on these studies, Th17 plays a protective role, mediated by their influence on the adaptive immune response, especially in Th1 responses. Similar to the other viruses seen so far, we do not know what the nature of these Th17 cells is, but they are probably non-pathogenic. With the data presented in the articles, the plasticity of Th17 seems to have a strong relevance as well.

### 4.4. West Nile Virus (WNV) and Adenovirus (Ad)

In addition to CD4 T lymphocytes, CD8 T lymphocytes play a fundamental role in the antiviral immune response. They act by eliminating infected cells, for example via perforin and granzyme, and by secreting cytokines that will act in an autocrine or paracrine manner. In relation to WNV, a neurotropic flavivirus that infects humans, Acharya et al. showed interesting data. Firstly, they observed that infected individuals had higher serum levels of IL-17A than healthy controls and that infection of human PBMCs with WNV led to the increased expression of IL-17A mRNA and cytokine secretion. Similar data were observed in mice. Using a murine model of WNV encephalitis, they demonstrated that *il-17a*^-/-^ mice were more susceptible to death, which they thought was related to greater permissiveness for the virus to invade the brain. Secondly, these authors established CD8 T cell coculture assays from mice with cells expressing a domain of the WNV envelope protein. In cocultures with CD8 T cells from *il17a*^-/-^ mice, CD8 T cells began to express smaller amounts of perforin, granzyme and FasL genes, compared with cells from wild-type mice [97].

Furthermore, confirming this relationship between Th17 cells and the response of CD8 T cells, using a murine model of hepatitis induced by Ad infection. Jie et al. demonstrated that Ad infection led to the expansion of IL-17A and IL-17F-producing intrahepatic T cells. The wild-type and *il17a*^-/-^ mice showed similar levels of inflammation and liver damage, while *il17f*^-/-^ mice developed a milder clinical condition, due to less inflammatory infiltrate in the hepatic tissue. These data suggested that there was a difference in the action of cytokines of the IL-17 family on CD8 T cells, in the context of viral infections [98].

### 4.5. Chikungunya (CHIKV), Dengue Virus (DENV) and Zika Virus (ZIKV)

One of the most studied arthritis-inducing viruses is CHIKV. There is evidence to suggest that CHIKV can replicate in the joints, thus stimulating an inflammatory response in the microenvironment that harms cartilage and bones [99]. Studies addressing in vitro infection have brought interesting data. Phuklie et al. demonstrated that CHIKV synoviocyte infection induces the production of inflammatory mediators such as IL-17, IL-6, IL-8, IFN-γ, MMPs and RANKL, which, as mentioned earlier, are possibly involved in the pathogenesis of rheumatoid arthritis [100]. Moreover, primary osteoblasts infected with CHIKV also show increased expression of RANKL and IL-6 [101].

Infection of mice deficient in B and T cells (*rag2*^-/-^) with CHIKV-induced persistent viremia, but without signs of inflammation in the joints as seen in wild-type mice, suggest that the role of the adaptive immune response is essential for joint involvement [101]. In this same model of infection, *cd4*^-/-^ animals showed improvement in joint swelling, thus confirming the need for CD4 T cells in this process [102]. In CHIKV-infected patients, those with a higher viral load had higher circulating levels of IL-6 in the first days of the disease, followed by an increase in IL-17 upon progression to the chronic phase, compared with patients with low viral load [103]. Moreover, Ng et al. suggested that IL-1β, IL-6 and RANTES would be markers of disease severity, since they showed elevated levels in critically ill patients, in comparison with healthy controls and individuals with mild conditions [104]. It has also been reported that the synovial fluid of patients infected with CHIKV presents elevated levels of IL-6 and IL-8 [99].

These studies demonstrate that an intense inflammatory profile with high levels of cytokines is crucial for the induction of harmful conditions caused by alphaviruses, such as arthritis. It is worth mentioning that these mediators are directly or indirectly related to the differentiation and effector function of Th17 cells. As seen in rheumatoid arthritis, IL-17 may induce RANKL expression and production of MMPs, which are directly related to destruction of cartilage and bone tissue [105,106,107,108,109]. Therefore, it is possible to suggest that Th17 would have an important role in inducing arthritis caused by CHIKV.

In DENV infection, patients who develop the severe form known as dengue hemorrhagic fever (DHF) have been found to present higher circulating levels of IL-17 than individuals with the mild form and healthy controls [110]. However, those who develop dengue shock syndrome also present IL-17 levels similar to those in DHF [111]. *Il-22*^-/-^ mice infected with DENV serotype 2 present greater disease severity, characterized by intense inflammation, liver damage and high production of IL-17A in the spleen and liver, compared with wild-type mice. This greater severity has also been found to be accompanied by higher mortality, increased serum levels of AST and ALT, accumulation of neutrophils and increased viral load in the liver. Interestingly, neutralization of IL-17A in *il-22*^-/-^ mice was found to reverse this situation, which suggests that the presence of IL-17A may be related to worse prognosis in DENV infection and that there is a negative regulatory loop between IL-17A and IL-22 [112,113].

A very recent study evaluated IL-17 and IL-17-producing cells in patients on different days and on different clinical outcomes. Interestingly, high serum concentrations of IL-17A and IL-22 have been associated with DHF. Consistent with the distorted immune response of IL-17 in patients with DHF, a high frequency of IL-17-producing CD4 + T cells was also observed. The authors concluded that IL-17A produced mainly by Th17 cells during secondary infections may play an important role during the “cytokine storm” and, consequently, contributes to dengue immunopathogenesis [114].

Regarding infection by ZIKV, it has already been observed that patients in the acute phase of this infection have high levels of cytokines relating to the differentiation and effector profile of Th17, such as IL-17, IL-1β and IL-6, compared with these same patients in the convalescent phase or healthy individuals [115]. In a study evaluating the levels of soluble mediators in asymptomatic individuals infected with DENV, ZIKV or WNV, it was observed that in all these infections, IL-17 levels were increased in comparison with uninfected individuals [116]. Naveca et al. evaluated a broad panel of cytokines along with daily measurements of viremia during the acute phase of ZIKV infection. Their data showed that, among other mediators, the levels of IL-17 and IL-1β, and to some extent, those of IL-6, accompanied peaks in viremia [117].

One of the most striking consequences of ZIKV infection is the condition of congenital Zika syndrome (CZS), which is characterized by a series of malformations in fetuses, including microcephaly. The immune response in situ in the brain tissue of infants with microcephaly who died shortly after birth and who came from mothers with a confirmed diagnosis of Zika during pregnancy was evaluated through immunohistochemistry tests in one study. It was seen that the brain in these babies had greater quantities of inflammatory infiltrate and higher numbers of cytokines from different profiles of T helper lymphocytes, including Th17, than in babies who died from other causes [118].

Note that few studies on the involvement of IL-17 in these arboviruses have been done, let alone on Th17 cells, mainly studies in patients. However, so far, Th17 seems to be more associated with the immunopathological response in arboviruses.

### 4.6. Viral Myocarditis

Myocarditis is an inflammation of the heart muscle, the myocardium. Myocarditis can resolve completely or result in chest pain, arrhythmia, heart failure or death. Viruses have been proposed to cause myocarditis, although in most cases, viruses are not identified or treatable. Among viruses, enteroviruses, adenoviruses, parvoviruses B19, Epstein–Barr virus (EBV), human herpesvirus (HHV) 6 and cytomegalovirus (CMV) are inducers of myocarditis, but picornaviruses, such as Coxsackievirus B3 (CVB3) and echovirus, are known as dominant pathogens [119,120].

Viral myocarditis can be a three-phase disease. In phase I, tissue damage is caused by viral replication in the absence of immune responses, so antiviral therapies are the ideal treatment. In phase II, immune and/or autoimmune antiviral responses contribute to immunopathology. In this phase, Th17 cells play important roles, and Th1 cells have also been proposed as the main inducers of immunopathology, although Th1 cells have also been shown to decrease viral replication [121]. Immune suppression is the most appropriate treatment at this stage. Dilated cardiomyopathy (phase III), the result of phases I and II, is characterized by cardiac structure and function remodeling and progresses independently of inflammation. In this phase, in some of the cases, the heart’s pump function is impaired, and the ventricles are dilated. Patients are treated with therapy for heart failure or even heart transplantation [122].

Studies by Yuan et al. demonstrated different actions of Th17 and Th2 cells in two different groups of patients, one with acute viral myocarditis (AVMC) and the other with dilated cardiomyopathy (DCM) with a history of AVMC. Mostly, Th17 cells as well as related cytokines and transcription factors were increased in AVMC, while Th2 cells, cytokines and transcription factors of their profile were increased in DCM. In addition, anti-cardiac IgG antibodies were found in most patients with AVMC and in half of the cases with DCM, accompanied by the increased expression of IL-17R in B cells. The authors concluded that Th17 cells helped B cells to produce anti-cardiac IgG in AVMC, and Th2 cells played an important role in mediating the humoral response only in the late stage of viral myocarditis [123].

The relationship between Th17 cells and CVB3 replication was demonstrated by Yuan et al. when the group infected BALB/c mice with CVB3 to establish AVMC models. The authors found an increase in viral replication, associated with an increased high frequency of splenic Th17 cells, serum IL-17 and cardiac IL-17 mRNA, accompanied by progressive cardiac AVMC lesions. Neutralization of IL-17 improved pathological cardiac changes, with a reduction in viral replication followed by decreases in cardiac inflammatory cytokines IL-17, TNF-α and IL-1β [124].

In this context, the blockade of Il-17 may represent a promising new therapeutic approach in the therapy of viral myocarditis diseases. In vitro experimental models also proved that the neutralizing anti-IL-17 antibody can inhibit the proliferation of B cells and the secretion of anti-adenine nucleotide translocator (ANT) autoantibodies [125]. Other findings suggest that IL-10-producing B cells may be a new therapeutic target for modulating the immune response in viral myocarditis, once IL-10-producing B cells negatively regulated the levels of T-bet and RORγt mRNA, decreasing the proportions of Th1 and Th17 cells to relieve inflammatory damage at an early stage of the disease [126]. In addition, specific IL-10-producing regulatory B cells pretreated with prostaglandin E2 (PGE2) expanded considerably and inhibited the differentiation of CD4 T cells into Th17 cells. In vivo, treatment with PGE2 significantly restricted the development of viral myocarditis [127]. Additionally, treatment with anti-CD80 monoclonal antibody regulates Th17 differentiation and expression of RORγt mRNA [128]. Finally, the anti-cholinergic inflammatory pathway attenuates the viral myocarditis inflammatory response and decreases the expression of cytokines in Th1 and Th17 cells [129].

Overall, the immunopathogenesis role of Th17 cells mediating viral myocarditis has been each more well stablished, and the blockade of IL-17 *per si* or IL-17-induced pathways represent a promising new therapeutic approach in the therapy of viral myocarditis diseases.

### 4.7. Viral Infection as a Trigger for Multiple Sclerosis

Multiple sclerosis (MS) is the most common autoimmune inflammatory disease of the central nervous system (CNS) [130]. It is characterized by the destruction of the protective myelin sheath of neurons, mediated by the infiltration of lymphocytes and other immune cells in the CNS [66]. The result is macroscopic lesions in the brain and progressive disability of the patient. MS can be subdivided into remitting–recurrent (RR), primary progressive (PP) or secondary progressive (SP) forms. The RR form is the dominant form at the onset of the disease and is characterized by acute clinical attacks followed by apparent disease stability. Symptoms can be relieved with various therapies, but in some patients, there is no beneficial effect, and the disease can progress to the form of SP. PP and SP remain difficult to treat and are also mechanically poorly understood [131].

The etiology of MS is still unknown, and among several factors, genetics contributes to the risk of developing MS. The main genetic risk factor is mapped in the class II and class I human leukocyte antigen (HLA) gene, whose main function is to present peptide ligands to CD4 + and CD8 + T lymphocytes, respectively. The MHC class II and I clusters contain polymorphic regions that are associated with protection against MS. Other genetic polymorphisms associated with MS are involved in immune responses, consistent with the concept that MS is an autoimmune disease induced by T cells [132]. Moreover, it is thought to require a provoking environmental insult such as a viral infection to trigger the disease [133]. Among viral infections, Epstein–Barr virus (EBV) shows the strongest association to MS induction [134]. In addition, antibodies against EBV, measles, rubella and herpes zoster have already been detected in the cerebrospinal fluid (CSF) of patients with MS, suggesting that the demyelination process may be accompanied by an antiviral immune response [135,136]. Neurotropic viruses can be a trigger for autoreactive immune responses. For example, viral infection by John Cunningham virus (JCV) activates cells present in the CNS, and infection of oligodendrocytes can cause cell death and demyelination [137].

Molecular mimicry is the mechanism most often discussed about how viruses can induce autoimmunity and MS. In general, autoreactive T cells are deleted in the thymus, stop responding at the periphery, or are even redirected to the Treg lineage that induces dominant immune suppression [138]. In fact, CD8 + T cell clones isolated from MS patients can be activated by basic myelin protein (MBP)- and the EBV EBNA-1 latency antigen (EBNA-1) peptide derivatives [139]. MS patients showed a selective increase in EBV-derived CD4 + T cell antigen in healthy virus carriers, but not to other EBV-encoded proteins [140].

The experimental autoimmune encephalomyelitis (EAE) is an animal model widely used to study neuroinflammation and MS, in the absence of viral infections [141]. An alternative model system of chronic progressive demyelinating disease induced by virus is the Theiler’s murine encephalomyelitis virus (TMEV) model [142]. Hou et al. reported that Th17 cells develop preferentially in vitro and in vivo in an IL-6-dependent manner following infection by the TMEV. The neutralization of IL-17 increases the elimination of the virus, increasing the lytic function of cytotoxic T cells and eliminating infected cells, leading to the prevention of the development of the disease. Thus, these results indicate a new pathogenic role for Th17 cells via IL-17 in persistent viral infection and its associated chronic inflammatory diseases [143].

Leukocyte migration into different tissues is controlled by specific adhesion molecules and chemokine receptors. The α4/β1 integrin appears to be critical in the migration of T cells to the CNS, while the identities of the relevant chemokine receptors are uncertain. One of the candidates is CCR6, which is stably expressed in human Th17 cells producing IL-17 [35]. In addition, we have the inflammatory chemokine receptors CXCR3 and CCR5, which are selectively expressed in Th1 and Th1/Th17 cells [144], and CCR7, which is also involved in the migration of T cells to the CNS and in MS [145]. Huppert et al. demonstrated that IL-17A induces the production of NADPH and reactive oxygen species (ROS) by brain endothelial cells, which trigger the cytoskeletal contraction machinery, causing the loss and disorganization of proteins in the narrow junctions and damaging the BBB. BBB function was recovered by inhibiting the formation of ROS or using IL-17A blocking antibodies, confirming the action of cytokine in this process [146].

Moreover, EAE model was fundamental for the identification of pro-inflammatory cytokines that can lead to pathogenic inflammation of the CNS. Mice not expressing IL-17A or neutralization of IL-17A using a monoclonal antibody leads to a decrease in the severity of EAE and even a delay in the induction of the disease. In addition, RORγt-deficient mice do not develop EAE [147]. Moreover, an important finding in autoimmunity was that IL-23 has a non-redundant pathogenic role in EAE and that IL-23 induces the maturation of Th17 cells. Interestingly, in viral encephalitis induced by neurotropic coronavirus in mice, IL-12, but not IL-23, increased morbidity, and this was associated with increased production of IFN-γ in T cells [148].

In the EAE model, different subsets of T cells, including Th1 and Th17 cells, could induce pathogenic neuroinflammation, although with different characteristics. Th1/17 cells that co-produce IFN-γ and IL-17 have high pathogenic potential and are also enriched in brain lesions in patients with MS [149]. However, neither IL-17 nor IFN-γ deficiency completely prevents EAE induction, whereas GM-CSF is necessary [150]. GM-CSF-producing T cells are also abundant in the CSF of MS patients [151].

An optional mechanism that could explain a pathogenic role of viral infections in MS is the bystander activation of autoreactive T cells. Tregs are responsible for inhibiting bystander activation [152], but several subsets of Tregs appear to have an impaired function in patients with MS [153]. Interestingly, patients with the RR form of MS have an expanded population of CCR6 + autoreactive T cells, which express CXCR3 and co-produce IL-17 and IFN-γ [154].

A high expression of the IL-17A gene in PBMCs and CFS in patients with MS has been demonstrated [155]. In addition, the expression of IL-17A would be associated with the number of lesions in the CNS and the severity of MS [155,156]. Another study showed the presence of lymphocytes with a Th1/Th17 profile in chronic active lesions of patients with MS [47,157]. Finally, Th1 lymphocytes from MS patients acquire the expression of *il23r*, *ccr6* and *rorγt*, suggesting that the co-expression of the Th1 and Th17 profile genes may be crucial for the induction of the disease [158]. Blood–brain barrier (BBB) dysfunction with increased endothelial permeability is another important feature in MS [159]. Th17 cells can cross BBB by secreting IL-17 and IL-22, which bind to their receptors expressed by endothelial cells and promote changes in the expression of tight junction proteins, such as ocludine and ZO-1 (from zonules occludens-1). In the same study, granzyme B secreted by Th17 induced neuronal death and contributed to the pathogenicity of the disease [159].

Finally, an interesting point to be mentioned is that in a medium where there is no TGF-β, but IL-1, IL-6 and IL-23, a subset of Th17 cells is generated with characteristics that are quite different from conventional Th17 cells. In this case, Th17 cells also express IL-33, a cytokine associated with inflammatory immune responses. In adoptive transfer experiments, the authors showed that these Th17 cells are very pathogenic and that in lesions of patients with multiple sclerosis, Th17 cells have these characteristics [160].

Thus, we can suggest that in the pathogenesis of MS and EAE, Th17 cells increase endothelial permeability and cross the BBB, facilitating the infiltration of inflammatory cells in the CNS. In addition, they secrete pro-inflammatory mediators in the tissue and mediate the inflammatory process and the immune response at the site, changing the function of neurons, microglia and astrocytes, leading to chronic demyelination, axonal damage and neuronal death.

## 5. Conclusions and Perspectives

**Figure 4:**
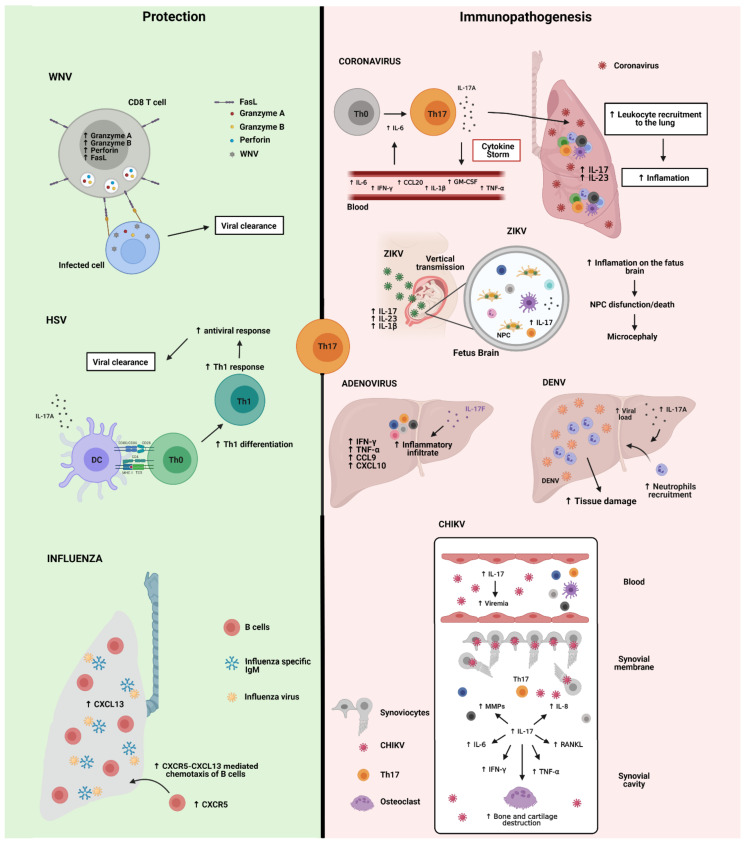
Th17 cells in the host’s immune response against viruses: protection or immunopathogenesis.

Recent advances have demonstrated the effector functions of Th17 cells in the host’s immune response against viruses. In this regard, deleterious effects from Th17 have also been observed in viral infections. These may be related to its ability to promote recruitment of cells with an inflammatory profile and to induce production of pro-inflammatory mediators by other cells at the same site. This, in turn, increases the inflammatory process and the damage to organ tissues. On the other hand, Th17 plays a key role in protecting and maintaining the mucous barrier. Thus, depending on the virus, the performance of Th17 cells and cytokines of the IL-17 family may increase the efficiency of antigen-presenting cells, the cytotoxicity of CD8 T cells or the antiviral activity of B cells. It is possible that viral dynamics influence the result of infection, i.e., elimination of the virus or establishment of persistent infection (Table 1 and Figure 4). Understanding the role of Th17 in viral infections can improve predictions of clinical outcomes and, even, patient treatment. After an extensive review of the literature, we realized that many published data addressed just the detection of circulating IL-17 levels and related cytokines in experimental models or sick patients, associating these levels with the clinical outcome. Few studies have evaluated the frequency and/or functionality of Th17 cells, as well as the subgroups of pathogenic Th17 and non-pathogenic Th17, or subpopulations of Th17 defined by the expression of markers, transcription factors and production of cytokines. Thus, it is necessary to stimulate lines of research that address further investigations of Th17 in viral infections, similarly to what has been done in SARS-CoV2 infection. In fact, since the emergence of the current pandemic, more than 80 scientific articles have been published, which already makes it possible to make available some reviews that exclusively address Th17 and COVID-19.

Severe acute respiratory syndrome coronavirus 2 (SARS-CoV-2); herpes simplex virus (HSV); Dendritic cells (DC); West Nile virus (WNV); Chikungunya (CHIKV); Dengue virus (DENV); Zika virus (ZIKV); Dengue hemorrhagic fever (DHF); Dengue shock syndrome (DSS); acute viral myocarditis (AVMC); adenine nucleotide translocator (ANT); Epstein–Barr virus (EBV); human herpesvirus (HHV-6); cytomegalovirus (CMV); Coxsackievirus B3 (CVB3); Varicella Zoster virus (VZV); Theiler’s murine encephalomyelitis virus (TMEV); blood–brain barrier (BBB); cerebral spinal fluid (CSF).

## Figures and Tables

**Table 1:** Th17 cells in viral infections—friend or foe?

Virus	Disease	Organism	Friend or Foe?	Evidence	Ref.
SARS-CoV2	COVID-19	Human	foe	Cytokine storm; polyfunctional Th1 and Th17 cells underrepresented in the repertoire of T cells reactive to SARS-CoV-2; lung tissue-resident memory-like Th17 cells; high frequency of Th17 cells and IL-17 levels in severe cases	[78,81,82,85]
Influenza Virus	Flu	Mice	friend	Role in the recruitment of B cells into the lungs; B1 cells differentiation and IgM production	[90,91]
HSV-2	Herpes	Mice	friend	Enhancement of DCs ability to induce a Th1 response	[94,95]
HSV-1	Herpes (RHL)	Human	foe	Increased Th17/Treg ratio and Th17 related cytokines in RHL patients	[96]
WNV	West Nile fever	Human	friend	Less permissiveness of viral invasion in the brain; activation of CD8 T cells	[97]
Ad	Hepatitis	Mice	foe	Expansion of IL-17A and IL-17F producing T cells in the liver; absence of IL-17F led to better clinical outcome	[98]
CHIKV	Chikungunya fever	Mice Human	foe	High levels of Th17 related cytokines in patients and CHIKV-infected cultures; high IL-17 levels involved in the progression to the chronic phase	[101,103]
DENV	Dengue fever	Mice Human	foe	High IL-17 levels in circulation and liver; high frequency of Th17 in DHF and DSS patients	[110,111,112,113,114]
ZIKV	Zika fever	Human	foe	High levels Th17-related cytokines in concomitant with viremia peaks; Th17 cytokines in the brain of microcephalic babies	[115,116,117,118]
Enteroviruses, adenovirus, parvoviruses B19, EBV, HHV-6, CMV, CVB3	Viral myocarditis	Human Mice	foe	Increased frequencies of Th17, IL-17 mRNA expression and Th17-related cytokines in AVMC patients and mice; induction of anti-ANT autoantibodies	[123,124,125,126,127,129]
EBV, measles, rubella, VZV, TMEV	Multiple sclerosis	Human Mice	foe	IL-17 inhibit activity of cytotoxic T cells; viral persistence; high levels of IL-17A in the CSF in MS patients; presence of Th1Th17 cells in brain lesions; migration of inflammatory cells to the brain through BBB disruption	[143,146,148,149,154,155,156,157,158,159,160]

## Data Availability

Not applicable.
